# A Multi-modality Approach Towards Elucidation of the Mechanism for Human Achilles Tendon Bending During Passive Ankle Rotation

**DOI:** 10.1038/s41598-018-22661-7

**Published:** 2018-03-12

**Authors:** Ryuta Kinugasa, Keigo Taniguchi, Naoto Yamamura, Mineko Fujimiya, Masaki Katayose, Shu Takagi, V. Reggie Edgerton, Shantanu Sinha

**Affiliations:** 1 Department of Human Sciences, Kanagawa University, Yokohama, Japan; 2 Computational Engineering Applications Unit, Advanced Center for Computing and Communication, RIKEN, Wako, Japan; 3 Department of Physical Therapy, Sapporo Medical University, Sapporo, Japan; 4 Department of Mechanical Engineering, The University of Tokyo, Tokyo, Japan; 5 Department of Anatomy, Sapporo Medical University, Sapporo, Japan; 6 Department of Integrative Biology and Physiology, University of California Los Angeles, Los Angeles, USA; 7 Department of Neurobiology, University of California Los Angeles, Los Angeles, USA; 8 Department of Neurosurgery, University of California Los Angeles, Los Angeles, USA; 9 Brain Research Institute, University of California Los Angeles, Los Angeles, USA; 10 Department of Radiology, University of California San Diego, San Diego, USA

## Abstract

The *in vitro* unconstrained Achilles tendon is nearly straight, while *in vivo* experiments reveal that the proximal region of the Achilles tendon, adjacent to Kager’s fat pad, bends ventrally during plantarflexion but remains nearly straight during dorsiflexion. Tendon bending is an important factor in determining the displacement of the foot compared to the shortening of the muscle fibers. The objective of this study was to elucidate the various mechanisms that could cause tendon bending, which currently remain unknown. Examination of Thiel-embalmed cadavers, with preservation of native articular joint mobility, revealed that the Achilles tendon still bent ventrally even when its surrounding tissues, including the skin surface, Kager’s fat pad, and distal portions of the soleus muscle were removed. Shear modulus and collagen fiber orientation were distributed homogeneously with respect to the longitudinal line of the tendon, minimizing their causative contributions to the bending. Given that tendon bending is not caused by either the nature of the deformations of the tissues surrounding the Achilles tendon or its physical properties, we conclude that it results from the geometric architecture of the Achilles tendon and its configuration with respect to the surrounding tissues.

## Introduction

The relationship between the action of the fibers and the joint that they influence is complex. For example, the ankle joint itself has at least twice the range of motion of that allowed by the shortening of the muscle fibers^1^. The shortening of the muscle fibers is amplified because of the pennate fiber orientation relative to the muscle’s line of action^2^, but this is not sufficient to account for the two-fold difference. An additional mechanical gain system (lever or gear) may operate directly on the extramuscular portion of a tendon itself, i.e., the Achilles tendon, occurring as a result of constraints on the direction of the tendon’s line of action by the presence of an obstruction, which manifests as a bend or inflection in the Achilles tendon.

A notable anatomical feature is that the *in vitro* Achilles tendon is nearly straight in the long axis even though both ends of the tendon are completely separated from the body and any tissues^3^. A simple mechanical explanation would be that an unconstrained Achilles tendon would straighten under tension. However, *in vivo* experiments have demonstrated that the Achilles tendon bends, even during relatively strong contractions^1,4–6^. This Achilles tendon bending was first reported and discussed by Maganaris *et al*.^1^. They implied that the triceps surae muscle/Achilles tendon would bend because of muscle thickening in the dorsoventral direction during plantarflexion. In an attempt to better understand the potential mechanisms behind the bending of the Achilles tendon and investigate its consequences, we recently published a series of reports on our experimental and modeling investigations. Our initial study^7^ speculated that an obstruction causes a bend in the Achilles tendon, which in turn acts as an amplifier for joint excursion. Using a simple geometrical model, this study showed that the mechanical consequence of such an obstruction would be to constrain the movement of the Achilles tendon and thereby amplify the displacement of the tendon’s end point compared to its start point. This speculation was subsequently successfully verified and observed *in vivo*, as a curvature of the Achilles tendon was seen when using an MRI spin-tag technique^5^. This study reported a significant dorsal curvature of the Achilles tendon in ankle plantarflexion during passive and active movement, which reduced tendon moment arms and favored high-velocity joint rotation. Our most recent study^8^ explained the creation of the curvature by reproducing it using sophisticated three-dimensional (3D) finite element modeling (FEM) and demonstrated that the fiber architecture of the triceps surae muscles, which play an important role in the final resultant motion of the muscle, impacted the magnitude and direction of Achilles tendon curvature during active plantarflexion. However, the anatomical structure and mechanisms that cause the bending remain unresolved.

The most obvious inherent difference between straight and bent Achilles tendons is the shape and extent of the deformation of tissues surrounding the tendon. The *in vivo* Achilles tendon is bounded by the soleus muscle inferiorly, calcaneal bone posteriorly, and the Kager’s fat pad (a mass of adipose tissue located within Kager’s triangle) anteriorly. The soleus muscle and the Kager’s fat pad are in line with each other and parallel to the Achilles tendon. During plantarflexion, the Achilles tendon behavior is likely to be affected by considerable differences in the elastic deformation of the soleus muscle and the Kager’s fat pad^9,10^. Therefore, in this study, we hypothesized that the different shapes and magnitudes of tissue deformation surrounding the Achilles tendon caused tendon bending during passive ankle rotation. To test our hypothesis, the anatomical location of tendon bending was first identified in living humans using MRI. Since active contraction would cause complex tendon behavior resulting from fiber orientation-induced muscle deformation^8^, paired with independent loading of each triceps surae muscle^11–13^; it was necessary to first simplify the problem and understand how the Achilles tendon bends when subjected to relatively simple passive movement, since even passive ankle rotation causes bending of the Achilles tendon^5^.

The objective of this study, therefore, was to elucidate the role that the following factors play in causing the bending of the human Achilles tendon during passive ankle rotation: (1) the different tissues surrounding the Achilles tendon, including the superficial skin surface, the Kager’s fat pad, and the soleus muscle. To examine the role of each, Thiel-embalmed cadavers, which exhibit preserved tissue compliance, flexibility, and joint mobility^14^ were studied and the tendon curvature measured while sequentially exteriorizing each of these tissues. (2) The internal mechanical and material characteristics of the Achilles tendon including (2-a) the shear modulus of the Achilles tendon using ultrasound supersonic shear imaging and, (2-b) the distribution of collagen fiber orientations within the Achilles tendon, which was determined using histochemical analysis and light microscopy. Failure of the above mechanisms to explain the bending of the Achilles tendon would require us to consider the third and final possible factor, namely, the structural dynamics of the tendon upon ankle rotation as determined by its geometric architecture and its configuration with respect to the surrounding tissues.

## Results

### MRI study – visualization and quantification of Achilles tendon curvature

**Figure 1:**
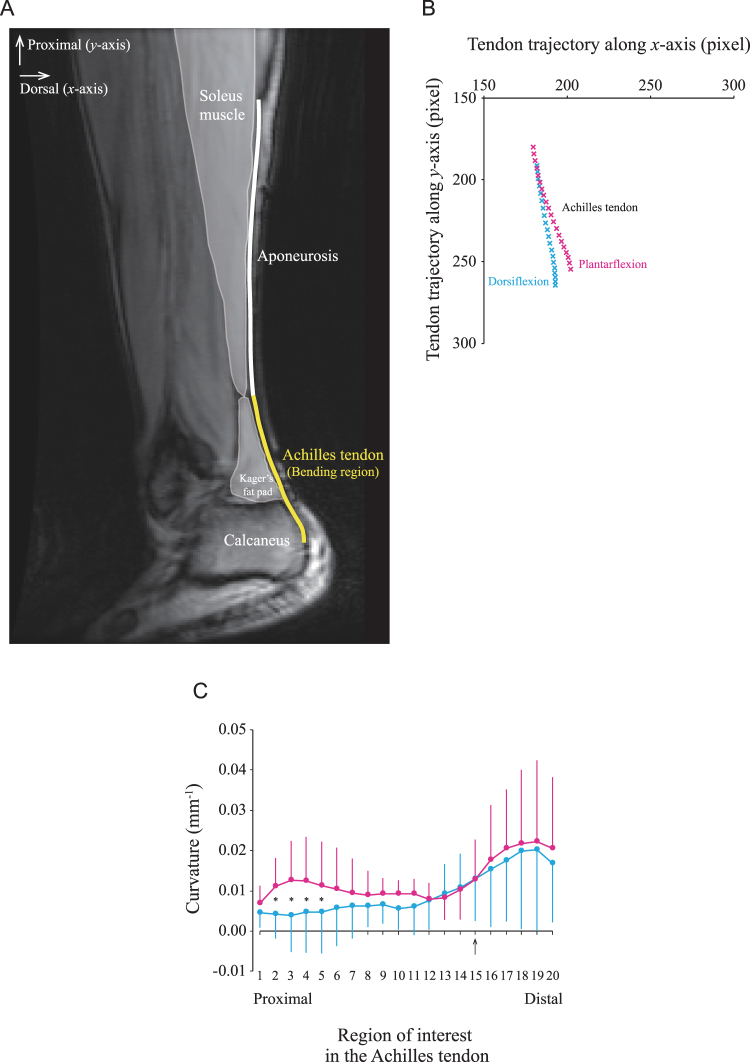
The trajectory and curvature along the entire length of the Achilles tendon during passive movement of the ankle. (**A**) Oblique sagittal magnitude MR image of the lower leg muscles. The thick white line is the aponeurosis proximally, and the thick yellow line is the Achilles tendon distally. The Achilles tendon is the line from the distal edge of the soleus muscle (start point) to the point of tendon insertion on the calcaneus, i.e., the inferior calcaneal tuberosity (end point). The line is almost straight in the aponeurosis region, but it is bent ventrally in the Achilles tendon region. The regions of interest were manually positioned at 20 points from the start to the end points of the Achilles tendon. (**B**) Scatter diagram of the *x* and *y* coordinates of the Achilles tendon with the ankle fully dorsiflexed (blue, cross symbol) and fully plantarflexed (pink, cross symbol). A one-way ANOVA was used to examine the difference in the entire length of the Achilles tendon between plantarflexion and dorsiflexion and to examine the differences in the vertical displacement and resultant vector between the start and end points of the Achilles tendon. Each data point represents the averaged values of 10 subjects. (**C**) The curvature of the entire Achilles tendon is compared between full ankle dorsiflexion and plantarflexion. The black arrow on the *X*-axis is an anatomic landmark corresponding to the same level as the boundary line between the most distal edge of the Kager’s fat pad and the superior calcaneal tuberosity. The difference in the curvature of the Achilles tendon was tested with a two-way ANOVA (2 ankle positions × 20 regions of interest). Each data point is represented by the mean and SD of n = 10. * indicates a significant difference (*P* < 0.05) between plantarflexion and dorsiflexion.

MRI measurements were taken to determine the curvature and behavior of the Achilles tendon when the ankle was passively moved from the –10° dorsiflexed position to the 20° plantarflexed position (Fig. 1A). The vertical displacement was significantly greater (*P* < 0.05) for the start point (13.3 ± 5.6 mm) for the Achilles tendon than for its end point (11.4 ± 5.5 mm) during plantarflexion. As expected, the end point of the Achilles tendon became substantially displaced perpendicular to the vertical axis of the tendon in contrast to that along the vertical axis of the tendon. However, the resultant vector was significantly greater (*P* < 0.05) for the end point (16.1 ± 6.2 mm) than for the start point (13.6 ± 5.5 mm), thereby amplifying the movement of the tendon’s end point compared to its start point (Fig. 1B). The length of the Achilles tendon elongated significantly (*P* < 0.05) from dorsiflexion (87.6 ± 26.4 mm) to plantarflexion (92.3 ± 25.6 mm). During dorsiflexion, the curvature was nearly zero from the proximal side to the middle along the long axis of the Achilles tendon. The curvature increased considerably from the middle to the insertion on the calcaneus, which may be attributed to the geometry of the calcaneus. This was also the case during plantarflexion. During plantarflexion, the Achilles tendon bent ventrally, particularly in the proximal portion. The curvature was significantly greater (*P* < 0.05) in regions of interest (ROIs) 2–5 during plantarflexion compared to dorsiflexion (Fig. 1C).

### Cadaveric study – the role of each of the surrounding tissues

#### Change in the curvature of the Achilles tendon with sequential removal of each of the surrounding tissues

**Figure 2:**
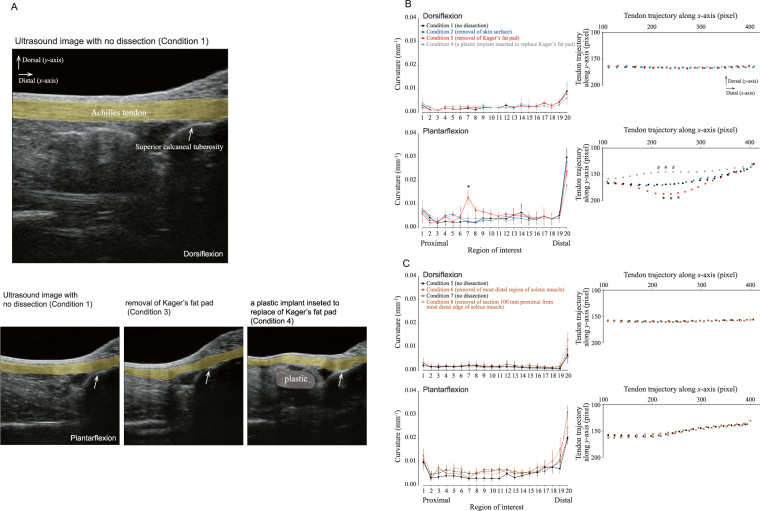
Effect of different dissections of the lower leg on the curvature and trajectory of the Achilles tendon in Thiel-embalmed human cadavers. (**A**) Representative sagittal longitudinal B-mode ultrasound images of the lower leg. The Achilles tendon is highlighted by a thick yellow line. The white arrow indicates the position of the superior calcaneal tuberosity, while the blue region indicates a plastic material (right bottom). (**B**) For the Kager’s fat pad dissection trial, the Achilles tendon curvature (left panel) and trajectory (right panel) is compared among four conditions (described in top-left sub-panel) during dorsiflexion and plantarflexion. The differences in the curvature and trajectory of the Achilles tendon were tested with a two-way (four dissection conditions × 20 regions of interest) ANOVA with repeated measures. Each data point represents the mean and SD of 7 legs. ^#^ indicates a significant difference (*P* < 0.05) between condition 4 vs. conditions 1, 2, and 3; * indicates a significant difference (*P* < 0.05) between condition 3 vs. conditions 1, 2, and 4. (**C**) For the soleus muscle dissection trial, the Achilles tendon curvature (left panel) and trajectory (right panel) is compared among four conditions during dorsiflexion and plantarflexion. The differences in the curvature and trajectory of the Achilles tendon were tested with a nonparametric Friedman two-way (four dissection conditions × 20 ROIs) ANOVA. Each data point represents the mean and SD of six subjects each for conditions 5–6 and conditions 7–8, respectively.

**Figure 3:**
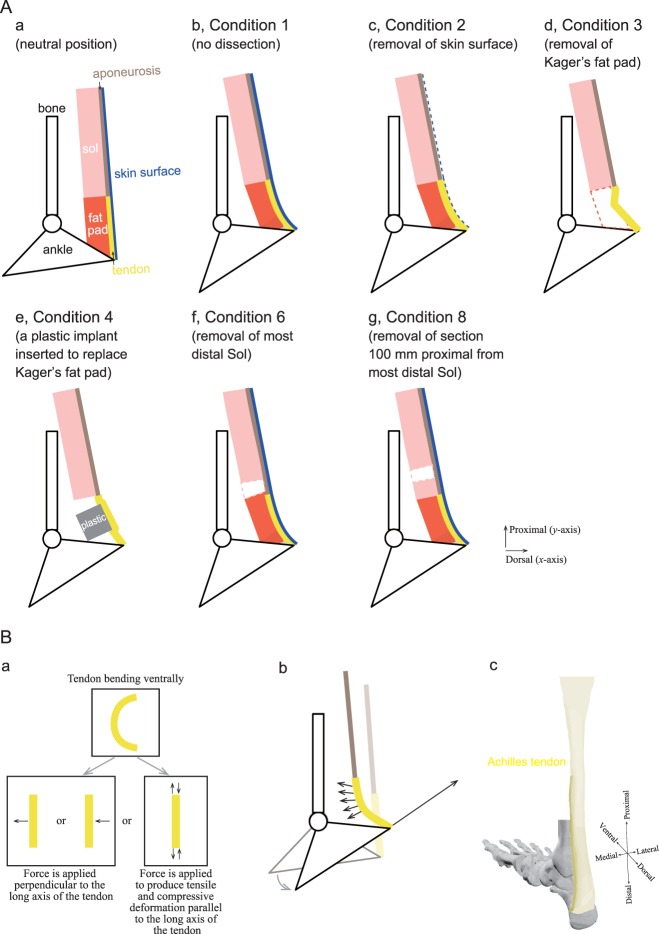
Schematic drawings to illustrate observed tendon bending patterns and a possible mechanism for Achilles tendon bending with passive ankle plantarflexion. (**A**) Diagram representing the observed tendon bending patterns. (a) The ankle is positioned in the neutral position. Red and pink indicate the Kager’s fat pad (fat pad) and soleus (sol) muscle, respectively. The Achilles tendon (tendon) (thick yellow line) is aligned in series with the aponeurosis (light brown line). (b) As the ankle is plantarflexed passively, the Kager’s fat pad constrains tendon movement in the ventral direction, but the skin surface (blue line) also seems to be constrained in the dorsal direction (Condition 1). (c) Even if the skin surface is removed (Condition 2), the magnitude of tendon bending is similar to that of Condition 1. (d), The tendon buckles significantly after removal of the fat pad (Condition 3). (e) Tendon buckling is not observed if a plastic material is implanted in place of the fat pad (Condition 4). (f and g) Removal of the most distal portion of the sol muscle (Condition 6) and the section of the sol muscle 100 mm proximal from the most distal edge of the sol muscle (Condition 8) does not alter the magnitude of tendon bending. (**B**) Schematic illustration of a possible mechanism for Achilles tendon bending. (a) Possible phenomenon of tendon bending in the ventral direction. The tendon will bend if a force is applied perpendicular to the long axis of the tendon or if a force is applied to produce tensile and compressive deformation parallel to the long axis of the tendon. (b) Tendon bending without the Kager’s fat pad, the sol muscle, aponeurosis, or skin surface. As the ankle plantarflexes, the calcaneus moves proximally as well as dorsally. These configurations seem to produce a force on the surrounding tissues and calcaneus. (c) A schematic of the geometrical properties of the Achilles tendon from one cadaver. The image was drawn from medial and posterior views. The length of the Achilles tendon along the proximodistal axis is long while the width along the mediolateral axis is narrow. Such geometrical properties provide lower flexural stiffness (i.e., deformable) in the dorsoventral direction rather than in the mediolateral direction.

Similar to the findings of the MRI study, the considerable curvature in the distal region may be attributed to the geometry of the calcaneus. During dorsiflexion, the Achilles tendon was nearly straight along the long axis of the tendon regardless of the condition, meaning that the curvature was nearly zero. In contrast, during plantarflexion, the Achilles tendon showed a gentle bend in the ventral direction before the dissection of any tissues (Condition 1) or removal of the skin surface (Condition 2). After removal of the Kager’s fat pad (Condition 3), the Achilles tendon buckled at the location of the Kager’s fat pad. As a result, the magnitude of the curvature and the movement in the ventral direction were significantly (*P* < 0.05) greater after removal of the Kager’s fat pad than (i) before dissection (Condition 1), (ii) after removal of the skin surface (Condition 2), as well as (iii) after a plastic implant was inserted to replace Kager’s fat pad (Condition 4) (Fig. 2B). On the other hand, the trajectory and curvature were not significantly different before tissue dissection (Conditions 5 and 7) or after removal of either region of the soleus muscle (Conditions 6 and 8) (Fig. 2C). The observed tendon bending patterns are schematically represented in Fig. 3A.

The intraclass correlation coefficient (ICC) value was 0.92 for the curvature measurement, and there was no significant difference in the mean values of the curvature between the two measurements. Therefore, the ultrasound measurement procedures yielded reproducible measurements of the curvature of the Achilles tendon.

#### Shear modulus measurement of the Achilles tendon

**Figure 4:**
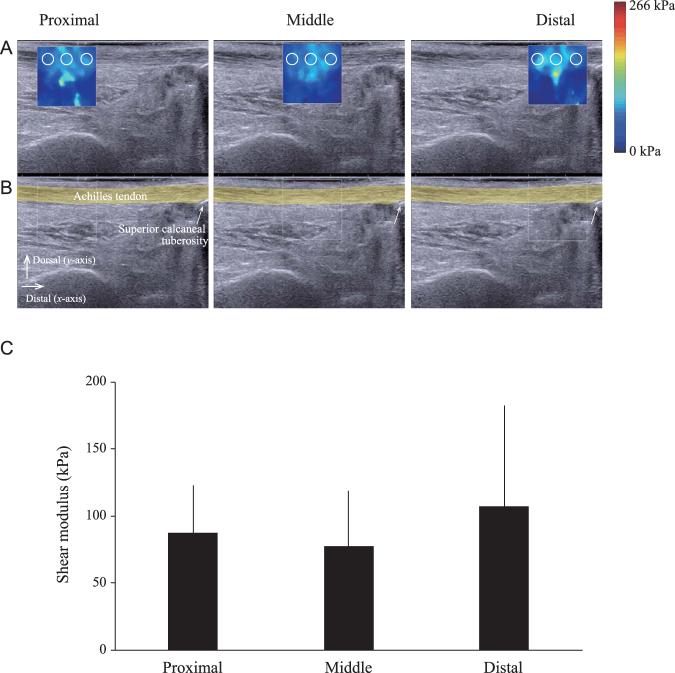
Ultrasound elastography demonstrating homogeneous shear modulus along the Achilles tendon. (**A**,**B**) Representative sagittal longitudinal ultrasound elastogram (Row **A**) and B-mode image (Row **B**) of the lower leg. The 15 × 15 mm region of interest (colored map at the top and white square line at the bottom) is positioned at the proximal, middle, and distal regions along the line of the Achilles tendon. For the sake of clarity of the Achilles tendon geometry, the echographic image without the color map, with the Achilles tendon in yellow, is depicted in the Row (**B**). The colored regions in the first row represent the shear elasticity map (stiffer areas are coded in red and softer areas in blue) with the color scale to the right of the figure. The mean values obtained in these three circular regions (2-mm diameter) are then averaged. In Row (**B**), the Achilles tendon is highlighted with a thick yellow line. The superior calcaneal tuberosity is indicated by a white arrow. (**C**) Shear modulus is compared between three different proximo-distal locations. A one-way ANOVA was used to examine the differences among the three locations of the shear modulus and indicated no significant differences. Each line represents the mean and SD of n = 6.

A typical example of the thickness measurement in the Achilles tendon was tested in a single sagittal image (Fig. 4A,B). The thickness of the Achilles tendon was 11.0 ± 0.5 mm for the proximal region, 11.2 ± 0.1 mm for the middle region, and 10.9 ± 0.2 mm for the distal region, indicating a mean thickness variation of 0.2 mm. The shear modulus of the Achilles tendon was 87.1 ± 36.0 kPa for the proximal region, 76.8 ± 42.3 kPa for the middle region, and 106.6 ± 75.6 kPa for the distal region. There was no significant difference in the shear modulus of the Achilles tendon among the three different locations (Fig. 4C).

The ICC value was 0.99 for the shear modulus measurement, and there was no significant difference in the mean values of the shear modulus between the two measurements. Therefore, the ultrasound measurement procedures yielded reproducible measurements of the shear modulus of the Achilles tendon.

#### Measurement of collagen fiber orientation and distribution

**Figure 5:**
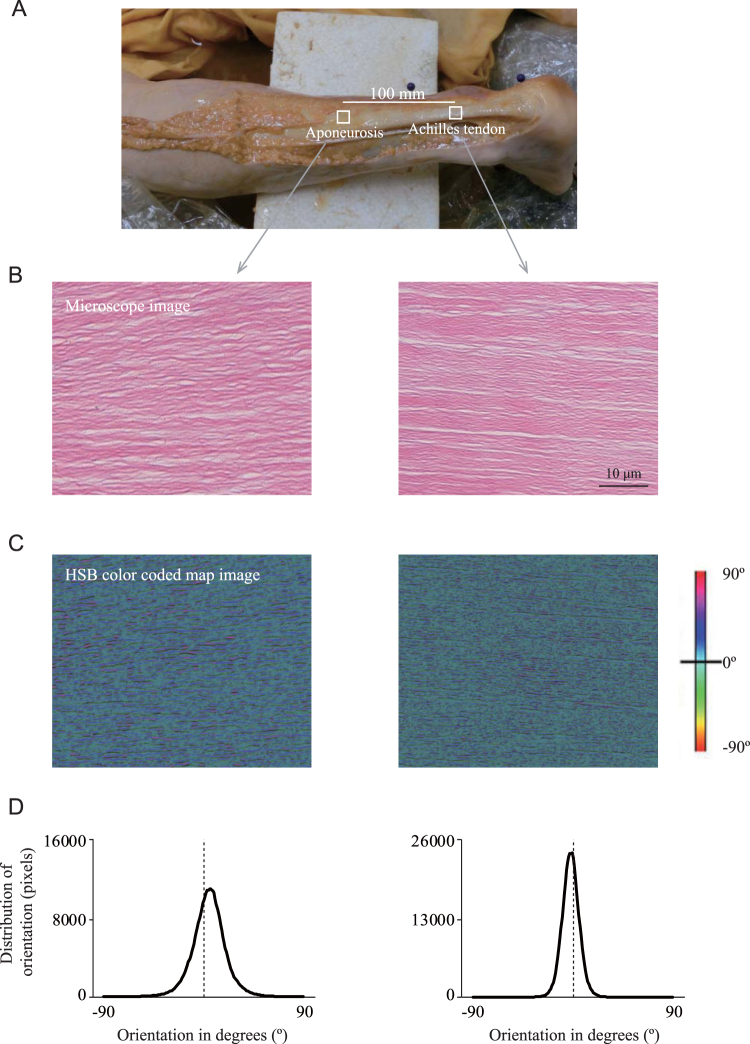
Collagen fiber orientation of the Achilles tendon and aponeurosis. (**A**) The Achilles tendon is exteriorized along with the proximal aponeurosis after removing the skin surface from the lower leg. (**B**) Typical longitudinal light micrograph (40× magnification) of the Achilles tendon and aponeurosis stained with hematoxylin show the wavy appearance of the collagen fibers. The left and right images show the microscope image from the Achilles tendon and aponeurosis, respectively. The bar indicates 10 μm. (**C**) Typical example of a hue-saturation-brightness (HSB) color-coded map, which shows the angles of the Achilles tendon and aponeurosis, respectively. (**D**) Typical orientation histogram of the collagen fibers at the Achilles tendon and aponeurosis, respectively. The orientation values in the histograms are weighted by the coherency values to give greater importance to the orientations that corresponded to elongated structures in the local neighborhood (n = 1).

Figure 5 shows the distribution of collagen fiber orientation in two different locations (the Achilles tendon and aponeurosis) of the samples prepared from the longitudinal plane sections (Fig. 5A,B). The obvious features of the Achilles tendon and aponeurosis were the wavy appearance of the collagen fibers with sparse fibroblasts interspersed within the interfibrillar space. This wavy pattern may be because of the resting tension that is intrinsic to the collagen fibers based on the periodic arrangement of the constituent amino acids^15^. Figure 5C illustrates typical examples of an HSB (Hue, Saturation, Brightness) color-coded map. These features are presented on a color map in HSB mode, with the hue representing orientation, saturation representing coherency, and brightness the source image. The map is clearly composed of sky-blue, blue, and green colors, suggesting that fiber orientation corresponded to a smaller angle of orientation. A sharp peak around 0° was observed in the Achilles tendon histogram rather than in the aponeurosis in this sample (Fig. 5D). In samples from four cadavers, the average value of collagen fiber orientation was measured as 2.41 ± 1.55° for the Achilles tendon and 2.38 ± 1.70° for the aponeurosis. The coherency was measured as 0.84 ± 0.07 for the Achilles tendon and 0.87 ± 0.04 for the aponeurosis. A coherency coefficient close to 1, represented as a slender ellipse, indicates a strongly coherent orientation of the local fibers in the direction of the long axis of the ellipse. Conversely, a coherency coefficient close to zero, represented geometrically as a circle, denotes no preferential orientation of the fibers. Hence, the overall pattern of collagen fibers in the Achilles tendon and aponeurosis appeared to be oriented nearly parallel to the longitudinal axis. We did not observe orthogonally oriented fibers relative to the longitudinal axis, significantly heterogeneous distributions, holes due to disconnected fibers, or damaged structures.

## Discussion

### Anatomical structure and mechanisms involved in tendon bending

In this study, we hypothesized that the different shapes and magnitude of deformations of the tissues surrounding of the Achilles tendon cause tendon bending. With the aim of proving or disproving the validity of this hypothesis, we examined the two possible factors that could cause the bending, namely, the different tissues surrounding the tendon and their mechanical and material characteristics. Our main finding was that even after removing the skin surface, the Kager’s fat pad, and the distal portions of the soleus muscle, the Achilles tendon remained bent and did not straighten during passive plantarflexion. Similarly shear modulus measurement and histochemical analysis of the tendon to examine the collagen fiber orientation showed that these factors could not contribute to the bending either. Therefore, as our hypothesis was proven null, we speculated on the final remaining mechanism, namely, the structural dynamics of the tendon upon passive ankle rotation.

The Achilles tendon seems to bend gradually rather than sharply at a particular point. The bend in the tendon is located adjacent to the Kager’s fat pad. Our cadaveric investigation revealed a difference in the measured tendon curvature with (Condition 1) and without the Kager’s fat pad (Condition 3) and after implantation of a plastic material (Condition 4), suggesting that the Kager’s fat pad may affect the extent of Achilles tendon bending. A further consequence was that path of the tendon bending changed depending on the stiffness of the Kager’s fat pad and, hence, ultimately determined the moment arm. Therefore, although the Kager’s fat pad is still widely considered to serve predominantly as a space filler^16,17^, it may also have a role in absorbing tendon deformity and the force generating capacity.

Heterogeneous distribution of tendon stiffness has been eliminated as one of the potential factors involved in tendon bending. Homogeneity of tendon stiffness is consistent with data from a study on human cadavers^18^. Although there is no detailed description of the embalming procedure in the methods section of this paper, their study obtained a similar value (around 100 kPa) of the shear modulus of the Achilles tendon at three different locations along the longitudinal axis in the neutral ankle position compared with our elastographic data from a Thiel-embalmed cadaver. However, it must be considered whether the tendon thickness varies along the longitudinal axis related to the shear wavelengths of the elastography^19,20^. Even if the tendon is assumed to have a constant thickness, this may not be entirely true as seen in the B-mode images presented in Fig. 4B. In the parallel configuration, a mean thickness variation of 0.2 mm was observed among the different ROIs. This thickness variability results in a mean relative deviation of 1% for the theoretical phase velocity dispersion curves obtained through the equation^20^. Therefore, a negligible variation of thickness is observed among the different ROIs in the Achilles tendon.

There does not appear to be any definitive anatomical structure causing the bending. If the collagen fiber orientation is a mechanism for the bending, it would require the collagen fibers to be orientated heterogeneously with respect to the line of the tendon at the location of the bending. In other words, the collagen fibers would need to be arranged in matrix-like mutually orthogonal directions at the location just above (proximal) and below (distal) the bend—such a heterogeneous orientation may facilitate bending of the tendon in the dorsoventral direction. In the present study, we prepared HE-stained samples for examination of the Thiel-embalmed human Achilles tendon and aponeurosis and to determine the collagen fiber orientation therein. Our HE-stained images indicated that in both the Achilles tendon and aponeurosis, undulating and bending collagen fibers are largely aligned parallel to the long axis of the tissue. A few fibers are interwoven with other fibers or have a spiral configuration, but a transverse collagen structure is not apparent (Fig. 5B), which is consistent with the findings of earlier studies^21,22^. Such a wavy orientation increases tendon elongation and helps release the energy stored within the tendon^23^. The heterogeneous collagen structure in the superficial-deep directions may also contribute to creating the bending factor, but future examination is needed to provide insight into the limits of these features.

Given its prominence in the literature, another factor that should be considered as possibly causative of tendon bending is the slackness of the tendon, which has been associated with a bent tendon^24,25^. Herbert’s group relied on the assumption that as muscle fascicle, aponeurosis, and extramuscular free tendons are classically considered to be in a series^26^, the slackness of these structures has been considered to occur at the same muscle-tendon length or joint angle^27,28^. They argue that the tendon becomes slack at short lengths^24^, but their indirect estimate of tendon length using an incomplete cadaveric tendon could be critical (the tendon was sectioned at a level 30 mm proximal to its insertion on the calcaneus)^29^. A few studies on humans have attempted to directly measure the tendon length^4,5,30,31^. There is a clear discrepancy in the change of Achilles tendon length during passive movement between our data presented here and other reports in the literature^5,30^. This may be explained by the differences in the measurement procedures of tendon length. It must be noted that measurement of tendon length in these previous studies does not reflect the entire length of the Achilles tendon but rather a portion of the tendon; they measured the tendon length until the level of the superior calcaneus tuberosity^5^ or at 80% of the overall length^30^. The former study represented the tendon as having an inflection at one point rather than a continuously bent tendon, and this may also contribute to the inconsistency. In contrast, our MRI study measured the entire length of the Achilles tendon from its start point to its end point, which is based on previous anatomical investigations where it was observed that the Achilles tendon is inserted into the inferior portions of the tuberosity beyond the superior tuberosity^32,33^. Such measurements clearly indicate that tendon bending is accompanied by elongation of the Achilles tendon length without any visible signs of slack in the sagittal MR image (Fig. 1A) or our 3D FEM (Supplementary Video S1). Although the tendon is presumed to expand along the transverse axis where its slackness occurs, direct MRI measurement revealed a shrinking of the Achilles tendon in the direction of the transverse axis during passive plantarflexion^30^. Taken together, the influence of tendon slackness on tendon bending would be minimal.

Given the above background, it becomes necessary to then consider the structural dynamics of the Achilles tendon as determined by its geometric architecture and its configuration with respect to its surrounding tissues as a possible mechanism for the bending. In order to cause ventral tendon bending in the direction indicated by our experimental data, one of the forces shown in Fig. 3B-a is necessary. The tendon will bend if a force is applied perpendicular to the long axis of the tendon or if a force is applied to produce tensile and compressive deformation parallel to the long axis of the tendon. As the ankle plantarflexes, the calcaneus moves proximally as well as dorsally. These configurations seem to produce a force on the surrounding tissues and calcaneus (Fig. 3B-b). The Achilles tendon is rather flat near its broad insertion to the calcaneus but becomes oval in the mid-region and then sheet-like as it courses proximally over the posterior surface of the soleus muscle^34^. Since the Achilles tendon is connected in series with the posterior aponeurosis, its length is relatively long in the proximodistal direction. In contrast, the width of the Achilles tendon is narrow in the mediolateral direction. Such geometrical architecture provides lower flexural stiffness (i.e., deformable) in the dorsoventral direction rather than in the mediolateral direction (Fig. 3B-c). Therefore, we concluded that geometric effects are likely to be responsible for ventral tendon bending.

This conclusion is further emphasized by the results of our 3D FE model (Supplementary Video S1). Using simulation with simple FE models, which included the Achilles tendon with the proximal aponeurosis, triceps surae muscles, ankle and knee joints but excluded the skin surface, subcutaneous fat, and the Kager’s fat pad, we demonstrated that although the length of the Achilles tendon remained consistent during passive ankle rotation, the Achilles tendon indeed bent in the ventral direction. Computer simulation is very effective in clarifying the involvement of factors that cannot be verified in physical experiments.

### Methodological considerations

Although there are conflicting reports regarding the influence of Thiel preservation on tissue mechanics, we believe that the collagen tissue in Thiel cadavers still retains sufficient histological and/or morphological integrity. Bone tissues from Thiel-preserved cadavers have been used in biomechanical testing without noticeable differences from fresh materials^35^; moreover, a histological analysis of Thiel-embalmed tendons has been reported to indicate a normal appearance^36^. There are also qualitative reports of preserved native tissue properties^36–40^. Conversely, recent biomechanical studies indicate reduced mechanical strength of bone after six months of direct immersion in a Thiel solution^41^, decreased collagen integrity in muscle from Thiel cadavers^14^, reduced tendon failure stress, and a trend towards a reduced elastic modulus^42^. This apparent discrepancy might be partly explained by variability in the rate of the diffusion of Thiel solution into the tendon tissues by the Thiel embalming protocol and the thickness of the tendons. This discrepancy requires further investigation.

### Conclusion

This study reveals the fundamental nature and implications of Achilles tendon bending using a multidisciplinary approach. Our investigations indicate that although the Kager’s fat pad serves to reduce the extent of the Achilles tendon curvature, neither constraints by surrounding tissues of the Achilles tendon nor heterogeneity with respect to the collagen fiber orientation and shear modulus in the Achilles tendon cause tendon bending; therefore, the geometric shape of the Achilles tendon itself must be involved. The tendon bending mechanism is important because the obstruction, which manifests as a bend of the tendon, is an anatomically inherent part of the normal internal design of the tendinous-musculoskeletal system. Elucidation of the mechanisms required for tendon bending will potentially help in the development of interventions required to improve efficient joint movement, in the design of artificial musculoskeletal structures, and in the creation of accurate modeling.

## Materials and Methods

### MRI study – visualization and quantification of Achilles tendon curvature

#### Subjects

Ten subjects (age: 25.1 ± 4.4 yr; height: 170.5 ± 11.0 cm; weight: 60.8 ± 8.8 kg, 6 men and 4 women) participated in the MRI study. None of the subjects had a history of previous or present Achilles tendon or lower limb injuries. All subjects gave written informed consent before inclusion in the study. Approval for the study was obtained from the ethical review board of the University of California San Diego. All experiments were performed in accordance with the Declaration of Helsinki.

#### Measurements

The anatomical location and movement of the Achilles tendon were determined using velocity-encoded phase-contrast (VE-PC) MRI. The details of this technique have been previously described^43^. Briefly, subjects were positioned in the foot-first supine position on the bed of a 1.5-Tesla MR scanner (Signa HDx, GE Medical Systems, Milwaukee, WI, USA). The right lower leg was fastened with Velcro tape to a custom-built MR-compatible foot-pedal device with a knee angle of 0°. The foot pedal was programmed to complete a rotational range of motion of 30°, from −10° (ankle dorsiflexion) to 20° (ankle plantarflexion) with a cyclic period of 2.86 s (21 cycles/min). The complete acquisition of VE-PC images required ~70 identical ankle joint rotations. The subject was instructed to fully relax as the ankle was passively plantarflexed and dorsiflexed using the computer-controlled servo motor of the foot-pedal device.

Oblique sagittal VE-PC images were acquired with a two-dimensional gradient echo PC sequence. The slice location was determined from the axial images and chosen to visualize the entire Achilles tendon. Velocity encoding of 100 mm/s was applied in all three directions (superior-inferior, anterior-posterior, and right-left) with four views per segment, a repetition time/echo time/flip angle of 13.3 ms/7.5 ms/20°, a 3-mm slice thickness, a 290-Hz receiver bandwidth/pixel, a 128 × 256 matrix size, a 160 × 320-mm field of view, and two averages. The retrospective gated mode was used to acquire 22 time-resolved frames during the rotation cycle. The temporal resolution of each frame was ~136 ms.

#### Data analysis

The ROIs were manually positioned at 20 points from the start to the end of the Achilles tendon using ImageJ software (Version 1.46r, National Institutes of Health, Bethesda, MD, USA). The start point of the Achilles tendon was defined as a boundary location between the most distal edge of the soleus muscle and the most proximal edge of the Kager’s fat pad, whereas the end point was at the inferior calcaneal tuberosity (Fig. 1A). The curvature (*k*) of the Achilles tendon ROI (*i*) was defined by

1 $${k}_{i}=\frac{2\parallel ({{\boldsymbol{p}}}_{i+1}-{{\boldsymbol{p}}}_{i})\times ({{\boldsymbol{p}}}_{i-1}-{{\boldsymbol{p}}}_{i})\parallel }{\parallel {{\boldsymbol{p}}}_{i+1}-{{\boldsymbol{p}}}_{i}\parallel \parallel {{\boldsymbol{p}}}_{i-1}-{{\boldsymbol{p}}}_{i}\parallel \parallel {{\boldsymbol{p}}}_{i+1}-{{\boldsymbol{p}}}_{i-1}\parallel }$$

where ${{\boldsymbol{p}}}_{i}$, ${{\boldsymbol{p}}}_{i-1}$, and ${{\boldsymbol{p}}}_{i+1}\,$are position vectors of ROI (*i*) and the neighboring ROI in the proximal-distal direction, as described by Kinugasa *et al*.^8^. The curvature was calculated over all the phases of the ankle rotation cycle. The entire length of the Achilles tendon was also determined as the length of the sum of the position vectors of the ROI from the start point to the end point of the Achilles tendon. The vertical displacement was calculated as $=yp-yd$ for the start point and end point of the Achilles tendon of the position vectors at the most ankle plantarflexed phase ($yp$) and at the most ankle dorsiflexed phase ($yd$). The resultant vector was calculated as $=\surd {(xp-xd)}^{2}+{(yp-yd)}^{2}$ for the start point and end point of the Achilles tendon of the position vectors at the most ankle plantarflexed phase ($xp,\,yp$) and the most ankle dorsiflexed phase ($xd,yd$).

### Cadaveric study – the role of each of the surrounding tissues

#### Cadavers

Twenty legs from 12 Thiel-embalmed cadavers (age at death 82.8 ± 7.6 yr; range 70–95 yr old; 8 women and 4 men) were used in our study. All the cadavers had been donated to Sapporo Medical University for anatomical research and education, and their use for research had been approved by the Medical Research Ethics Committee at Sapporo Medical University. Informed consent was obtained from the individual and their families before their death. Cadavers with obvious deformities or signs of surgical intervention in their ankle joints were excluded. All experiments were performed in accordance with the Declaration of Helsinki and Guidelines for Cadaver Dissection in Education and Research of Clinical Medicine by Japan Surgical Society and Japanese Association of Anatomists.

#### Experimental design

**Figure 6:**
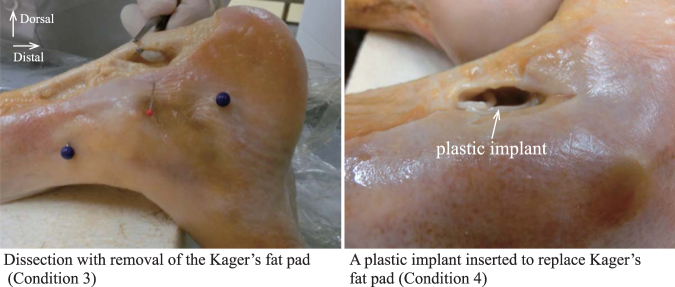
Examples of dissection with removal of the Kager’s fat pad (right) and implantation of a plastic material into the Kager’s fat pad (left). The right image (medial view of a right lower leg) and left image (lateral view of a right lower leg) were obtained after removal of the Kager’s fat pad and after a plastic implant was inserted to replace Kager’s fat pad, respectively.

The cadaver study was divided into 2 trials: Kager’s fat pad dissection and soleus muscle dissection. Both trials measured whether the Achilles tendon trajectory and curvature were preserved under 8 conditions with 1) no dissection, (2) removal of the skin surface, (3) removal of the Kager’s fat pad, (4) a plastic implant inserted to replace the Kager’s fat pad, (5) no dissection, (6) removal of the most distal region of the soleus muscle, (7) no dissection, (8) removal of the section of the soleus muscle 100 mm proximal from the most distal edge of the soleus muscle. Conditions 1–4 were the Kager’s fat pad dissection trial, and conditions 5–8 were the soleus muscle dissection trial. Conditions 1–4 were performed in 6 legs, while conditions 5–6 and conditions 7–8 were performed in 7 different legs each. For Condition 2, the skin surface was removed from the calcaneus bone to the proximal part of the myotendinous junction between the soleus muscle and the Achilles tendon. For Condition 3, only the entire Kager’s fat pad was carefully removed (Fig. 6) after confirming its location by an ultrasound scanner (α7, Hitachi-Aloka Medical, Tokyo, Japan). Care was taken not to damage the Achilles tendon or the soleus muscle with the surgical tools. For Condition 4, an extremely stiff plastic material (more than 800 kPa), was used for the implant to replace the Kager’s fat pad. These dissections left the subcutaneous fat, fascia, and the soleus muscle intact. For Conditions 6 and 8, an approximately 20 × 20 mm section of the skin surface, the subcutaneous fat, and the collagenous-based fascia had to be opened medially to reach the soleus muscle. Subsequently, either the most distal region of the soleus muscle or the section 100 mm proximal from the most distal edge of the soleus muscle was removed. Only the muscle component was carefully removed from the soleus posterior aponeurosis because the distal compartment of the soleus muscle was wrapped and interfaced with the connective tendinous tissues. Care was taken not to damage the Achilles tendon or the soleus posterior aponeurosis with the surgical tools. These dissections left the Kager’s fat pad intact. All of these surgeries were performed with the leg attached to the main body of the cadaver specimen.

#### Measurements and data analysis

(i) Change in the curvature of the Achilles tendon with sequential removal of each of the surrounding tissues: An ultrasound scanner equipped with a 50-mm linear-array probe (7.5 MHz) fitted with an attachable water pad was used to produce an oblique sagittal plane image. The cadaver was laid prone on the surgical table with approximately 60° hip and 0° knee angles. The ultrasound probe was positioned on the surface of the skin such that the most bent portion of the Achilles tendon in the ankle plantarflexion position and superior calcaneus tuberosity were clearly visible within the plane (Fig. 2A). An investigator manually moved the cadaver’s ankle passively from the most plantarflexed position to the most dorsiflexed position with a cyclic period of approximately 2 sec. This measurement was conducted 2–4 times. Ultrasound images were acquired at 30 Hz and stored on the hard drive of the scanner.

The ROIs were manually positioned at 20 points from the distal side of the superior calcaneal tuberosity to the most proximal edge of the ultrasound image using ImageJ software (Version 1.46r, National Institutes of Health, Bethesda, MD, USA). Again, the curvature (*k*) of the Achilles tendon ROI (*i*) was defined by the above-mentioned equation [1], and the curvature was calculated over all phases of the ankle rotation cycle. The trajectory and curvature were measured 2–4 times and averaged.

(ii) Shear modulus measurement of the Achilles tendon: An Aixplorer ultrasonic scanner (Supersonic Imagine, Aix en Provence, France) was also used to measure the shear modulus of the Achilles tendon^18–20^. Six cadaveric legs were used in this measurement after dissection of the skin surface (above-mentioned Condition 2). A 50-mm linear-array probe (8 MHz) was aligned carefully with the longitudinal axis of the Achilles tendon. The scanner was set at the supersonic shear imaging mode. The central point of the ultrasound probe was placed on the Achilles tendon, which corresponded to the same location as the most bent location of the Achilles tendon in the ankle plantarflexed position, and carefully aligned with the longitudinal direction of the tendon (Fig. 2A). The ankle angle was set at around 20° during measurement. The measurement was conducted twice.

For the analysis of the shear modulus, the ultrasound scanner software allowed us to measure the mean shear elasticity values obtained from three circular regions (2-mm diameter) of the Achilles tendon. The shear modulus was then calculated at three different locations (proximal, middle, distal) along the Achilles tendon. The shear modulus was measured twice and averaged.

(iii) Measurement of collagen fiber orientation and distribution: Collagen fiber samples were dissected from two locations – the Achilles tendon and the aponeurosis – after the successful completion of the biomechanical study described above (Fig. 5A). The sample from the location of the inflection point was from the Achilles tendon, and the sample from the location near the soleus muscle at its most distal edge was from an aponeurosis of the posterior compartment of the soleus muscle. To examine the histological features of the collagen fibers in the Achilles tendon and aponeurosis from four cadavers, these samples were dehydrated through a graded alcohol series and embedded in paraffin before being cut into sections (3 µ) along the longitudinal plane. The sections were stained with hematoxylin-eosin (HE) and observed using polarized light microscopy (Eclipse 50iPOL, Nikon, Tokyo, Japan).

The local collagen fiber alignment was determined with the ImageJ plug-in OrientationJ (Version 19.11.2012., available online at http://bigwww.epfl.ch/demo/orientation/)^44^. We analyzed the local tensor structure and matrix representatives of partial derivatives. The collagen fiber orientation and coherency of the Achilles tendon and aponeurosis were measured. To avoid sample bias, each section was averaged over 9 different regions: anterior-medial, anterior-central, anterior-lateral, central-medial, central-central, central-lateral, posterior medial, posterior-central, and posterior-lateral.

### Statistical analysis

Values are presented as the mean and standard deviation. For the MRI study, differences in the curvature and trajectory of the Achilles tendon were tested with a two-way (two ankle positions × 20 ROIs) ANOVA. A one-way ANOVA was used to examine the difference in the entire length of the plantarflexion. For the cadaver study, differences in the curvature and trajectory of the Achilles tendon were tested with a two-way (four dissectioAchilles tendon between plantarflexion and dorsiflexion and to examine the differences in the vertical displacement and resultant vector between the start and end points of the Achilles tendon during n conditions × 20 ROIs) ANOVA with repeated measures for the Kager’s fat pad dissection trial and a nonparametric Friedman two-way (four dissection conditions × 20 ROIs) ANOVA for the soleus muscle dissection trial. Each condition was not truly independent but had a within-subjects factor, i.e., sometimes a subject may have undergone several measurements. In a broader sense, the term repeated measure is a synonym for a within-subject factor^45^. A one-way ANOVA was used to examine the difference in the shear modulus among the three locations. Bonferroni or Dunnett tests were used for post hoc analysis where appropriate. The reproducibility of curvature and shear modulus measurements on ultrasound was assessed by an ICC, which estimates the within-investigator reliability of measures, and the Student’s paired *t*-test. The level of statistical significance was set at *P* < 0.05.

## Electronic supplementary material

Supplementary Video S1

Supplementary Video S1
