# Supplementary material for: A Multi-modality Approach Towards Elucidation of the Mechanism for Human Achilles Tendon Bending During Passive Ankle Rotation
Source: Sci Rep. 2018 Mar 12;8:4319. doi: 10.1038/s41598-018-22661-7 (PMC5847516; doi:10.1038/s41598-018-22661-7)
Supplement: Supplementary file 1 — Supplementary Video S1 [file 41598_2018_22661_MOESM1_ESM.doc]

**SUPPLEMENTAL INFORMATION**

A Multi-modality Approach Towards Elucidation of the Mechanism for Human Achilles Tendon Bending During Passive Ankle Rotation

Ryuta Kinugasa, Keigo Taniguchi, Naoto Yamamura, Mineko Fujimiya, Masaki Katayose, Shu Takagi, V. Reggie Edgerton, Shantanu Sinha

**Bending of the Achilles tendon during passive ankle rotation using three-dimensional finite element modeling (Supplementary Video S1)**

Experimental data and finite element (FE) model from our previous study were used (ref. 46). Briefly, the 3D FEM of the lower limb was constructed based on structural MRI of a single healthy subject. The MRI was segmented to define the anatomical structures, and a finite element mesh was fit to each structure. Our model included the Achilles tendon with the proximal aponeurosis, triceps surae muscles, ankle, and knee joint but excluded the skin surface, subcutaneous fat, and the Kager’s fat pad. FE meshes were constructed including 8 hexahedral elements and 76,862 nodes. The meshes consisted of two materials – triceps surae muscles and Achilles tendon with proximal aponeurosis – connected rigidly by coincident nodes. Muscles and tendons were represented using a nearly incompressible, hyperelastic, transversely isotropic constitutive model. Bones were represented as rigid bodies. The Achilles tendon-calcaneal bone interface was represented by rigidly attaching the element faces of the tendon to the bone surface. The simulation was performed with the ankle angle ranging from 0º to 25º using a software which first developed as a freely available V-Biomech (ref. 47, available online at http://vcad-hpsv.riken.jp/en/release_software/V-Biomech/). Ankle plantarflexion was done with passive movement of the calcaneus, and the motion of the Achilles tendon was captured.

**Supplementary References**

46.  Yamamura, N. *et al*. Effect of tendon stiffness on the generated force at the Achilles tendon – 3D finite element simulation of a human triceps surae muscle during isometric contraction. *JSME* **9**, 13–00294 (2014).

47.  Alves, J. L., Yamamura, N., Oda, T., Teodosiu, C. *Numerical simulation of musculo-skeletal systems by V-Biomech*. Proceedings of Computer Methods in Biomechanics and Biomedical Engineering, p. 43–48 (2010).
